# Quantifying uncertainty in microbiome-based prediction using Gaussian processes with microbial community dissimilarities

**DOI:** 10.1093/bioadv/vbaf045

**Published:** 2025-03-11

**Authors:** Asahi Adachi, Fan Zhang, Shigehiko Kanaya, Naoaki Ono

**Affiliations:** Graduate School of Science and Technology, Nara Institute of Science and Technology, Ikoma 630-0192, Japan; Graduate School of Science and Technology, Nara Institute of Science and Technology, Ikoma 630-0192, Japan; Graduate School of Science and Technology, Nara Institute of Science and Technology, Ikoma 630-0192, Japan; Data Science Center, Nara Institute of Science and Technology, Ikoma 630-0192, Japan; Graduate School of Science and Technology, Nara Institute of Science and Technology, Ikoma 630-0192, Japan; Data Science Center, Nara Institute of Science and Technology, Ikoma 630-0192, Japan

## Abstract

**Summary:**

The human microbiome is closely associated with the health and disease of the human host. Machine learning models have recently utilized the human microbiome to predict health conditions and disease status. Quantifying predictive uncertainty is essential for the reliable application of these microbiome-based prediction models in clinical settings. However, uncertainty quantification in such prediction models remains unexplored. In this study, we have developed a probabilistic prediction model using a Gaussian process (GP) with a kernel function that incorporates microbial community dissimilarities. We evaluated the performance of probabilistic prediction across three regression tasks: chronological age, body mass index, and disease severity, using publicly available human gut microbiome datasets. The results demonstrated that our model outperformed existing methods in terms of probabilistic prediction accuracy. Furthermore, we found that the confidence levels closely matched the empirical coverage and that data points predicted with lower uncertainty corresponded to lower prediction errors. These findings suggest that GP regression models incorporating community dissimilarities effectively capture the characteristics of phylogenetic, high-dimensional, and sparse microbial abundance data. Our study provides a more reliable framework for microbiome-based prediction, potentially advancing the application of microbiome data in health monitoring and disease diagnosis in clinical settings.

**Availability and implementation:**

The code is available at https://github.com/asahiadachi/gp4microbiome.

## 1 Introduction

Human-associated microorganisms form diverse and complex microbial communities. These microbial communities, along with their activities, are collectively known as the microbiome (Berg *et al.* 2020). The human microbiome exists in various sites of the body, including the gastrointestinal tract, oral cavity, and skin. Advances in next-generation sequencing and bioinformatics have significantly expanded our understanding of the human microbiome. According to Fan and Pedersen (2021), the human microbiome plays important roles in biological processes such as metabolism and immunity. Moreover, despite wide variation in microbial composition among individuals, healthy microbiomes typically exhibit high diversity, richness, and stability. Another study has highlighted its dynamic nature and rapid response to environmental changes, diet, and host factors, suggesting a significant impact on human health (Hou *et al.* 2022).

The human microbiome is associated with the health and disease of the human host. An imbalance in the microbiome is known as dysbiosis, which is characterized by the loss of beneficial microorganisms, expansion of pathogenic microorganisms, and a reduction in overall microbial diversity (Petersen and Round 2014). Microbial dysbiosis is related to the development and progression of various diseases, including inflammatory bowel disease (IBD), obesity, diabetes mellitus, cancer, brain disorders, and cardiovascular diseases (Hou *et al.* 2022). These associations between the microbiome and human health underscore the potential of microbiome research to understand disease mechanisms and suggest that detailed examinations of microbial communities could provide valuable insights for disease diagnosis and precision medicine (Berg *et al.* 2020).

The human microbiome has recently been used to predict host health and disease (Correa-Garcia *et al.* 2023). Machine learning models have been developed to harness phylogenetic, high-dimensional, and sparse microbial data. These models typically use microbial abundance data, represented as operational taxonomic units (OTUs) or amplicon sequence variants (ASVs) derived from 16S rRNA gene amplicon sequences, to predict biological or clinical outcomes. Random forests and lasso are commonly used for these predictions (Asnicar *et al.* 2024). Convolutional neural networks (CNNs) and kernel methods have also been utilized to incorporate microbial phylogenetic information (Sharma *et al.* 2020, Wang *et al.* 2021, Li *et al.* 2023, Xu *et al.* 2024, Wang *et al.* 2024). These microbiome-based prediction models have potential for improving disease risk assessment, tracking disease progression, and predicting severity (Liu *et al.* 2024).

Quantifying uncertainty in prediction is essential for applying machine learning models in real-world healthcare settings. Uncertainty estimates help healthcare providers make informed decisions (Kompa *et al.* 2021). For example, additional examinations may be requested, or outliers may be detected, based on the level of uncertainty in the predictions. Furthermore, uncertainty estimates are important for dataset shifts (Ovadia *et al.* 2019). A dataset shift occurs when the distribution of observed data deviates from the original training data. In such cases, considering low predictive confidence helps in accurately assessing risk, identifying performance degradation, and avoiding premature decision-making. Thus, measuring uncertainty and confidence in predictions is important for the effective application of microbiome-based prediction models in clinical settings.

However, traditional machine learning models typically provide point predictions without considering predictive uncertainty. Fully Bayesian models handle uncertainty through posterior distributions but require significant computational resources. To balance accuracy and computational efficiency in uncertainty quantification, point prediction methods are often extended to include predictive uncertainty. For example, quantile regression forests (Meinshausen 2006) extend random forests for quantile predictions, whereas NGBoost (Duan *et al.* 2020), a gradient boosting method, uses natural gradients for probabilistic regression. In addition to these tree-based methods, Monte Carlo dropout (Gal and Ghahramani 2016) estimates uncertainty through dropout in neural networks (NNs). Deep ensembles (Lakshminarayanan *et al.* 2017) improve uncertainty estimates by combining the output distributions of multiple independently trained NNs, thereby offering a scalable alternative to Bayesian methods. Although NN-based approaches are practical and scalable for estimating uncertainty, they are prone to overfitting, which may affect the reliability of their uncertainty estimates. It remains crucial to assess predictive models from a probabilistic perspective using methods grounded in a stronger theoretical understanding of probability distributions.

Gaussian processes (GPs; Rasmussen and Williams 2005) provide a sophisticated probabilistic framework for addressing uncertainty. Viewed as a Bayesian nonparametric approach based on kernel methods, GPs yield predictions as posterior probability distributions, offering both a mean estimate and a measure of uncertainty, typically represented as variance. These models are well-suited for capturing complex nonlinear patterns in data and are effective in high-dimensional spaces. GPs are also resistant to overfitting, even with limited datasets, and offer flexibility in incorporating domain-specific knowledge through the design of kernel functions. These advantages have led to their widespread applications across various fields, including bioinformatics and computational chemistry (Kalaitzis and Lawrence 2011, Deringer *et al.* 2021). The theoretical and practical aspects of GPs make them essential for developing reliable models.

In this study, we propose a method for achieving more accurate uncertainty estimates in microbiome-based prediction models. We developed a model that integrates microbial community dissimilarities into the kernel functions of GPs. Our results demonstrate that the proposed method enables more accurate uncertainty quantification while maintaining or improving point prediction accuracy compared with existing machine learning methods. This approach represents a significant advancement in developing reliable microbiome-based prediction models.

## 2 Methods

### 2.1 Gaussian processes

A GP (Rasmussen and Williams 2005) is a probability distribution over functions f(x), where any finite number of function values follow a joint Gaussian distribution. It is fully specified by its mean function m(x) and kernel (or covariance) function $k(x,x^{\prime })$, as described in (1). In this study, we employed a zero mean GP prior for the latent function.


(1) $$f(x)∼GP\left(m(x),k(x,x^{\prime })\right).$$

In regression models that assume additive Gaussian noise with variance $\sigma_{n}^{2}$, the observed values are generated by adding Gaussian noise to the latent function values of the GP. Thus, the likelihood function is modeled as a normal distribution centered at the latent function values with variance $\sigma_{n}^{2}$. The predictive distribution for a single test input $x_{*}$ follows a normal distribution, which can be computed analytically as follows:


(2) $$p(y_{*}|X,y,x_{*})=N\left(\mu_{*},\sigma_{*}^{2}\right)$$

(3) $$\mu_{*}=k_{*}^{⊤}{\left(K+\sigma_{n}^{2}I\right)}^{-1}y$$

(4) $$\sigma_{*}^{2}=k_{**}+\sigma_{n}^{2}-k_{*}^{⊤}{\left(K+\sigma_{n}^{2}I\right)}^{-1}k_{*},$$

where K is the covariance matrix on the training inputs X, $k_{*}$ is the covariance vector between the test input $x_{*}$ and training inputs X, and $k_{**}=k(x_{*},x_{*})$.

### 2.2 Microbial community dissimilarities and kernel functions

In microbial community ecology, measuring the dissimilarities between communities plays a critical role in understanding microbial structures. Various dissimilarity indices or distances, known as β diversity metrics, have been developed to assess community dissimilarities. These indices can be broadly grouped into four categories based on two characteristics: whether they are quantitative or qualitative and whether they incorporate phylogenetic information (Lozupone and Knight 2008, Peterson *et al.* 2024). Quantitative measures use abundance data, whereas qualitative measures use only presence/absence data. To explore these categories, we selected a representative dissimilarity index from each category and applied them to the GP kernel function. The indices used were Jaccard (5), Bray–Curtis (6, Bray and Curtis 1957), unweighted UniFrac (7; Lozupone and Knight 2005), and weighted UniFrac distances (8; Lozupone *et al.* 2007). The distance between the microbial communities A and B is calculated as follows:


(5) $$d_{\text{JA}}(A,B)=1-\frac{\left|A\cap B\right|}{\left|A\cup B\right|},$$

(6) $$d_{\text{BC}}(A,B)=\frac{\sum_{i=1}^{n}|A_{i}-B_{i}|}{\sum_{i=1}^{n}\left(A_{i}+B_{i}\right)},$$

(7) $$d_{\text{UU}}(A,B)=\frac{\sum_{j=1}^{m}b_{j}|I(A_{j}>0)-I(B_{j}>0)|}{\sum_{j=1}^{m}b_{j}},$$

(8) $$d_{\text{WU}}(A,B)=\frac{\sum_{j=1}^{m}b_{j}|A_{j}-B_{j}|}{\sum_{j=1}^{m}b_{j}(A_{j}+B_{j})},$$

here, $A_{i}$ and $B_{i}$ are the relative abundance of species i in A and B, respectively, $b_{j}$ represents the length of a branch j in the phylogenetic tree, and I(·) is the indicator function.

Next, we designed kernel functions of the GP using these dissimilarity indices, inspired by the radial basis function (RBF), or squared exponential covariance function, which is commonly employed in GP. The RBF kernel quantifies distances between data points based on Euclidean distances. In a similar manner, kernel functions were developed based on the dissimilarity indices, with the length scale l and the output scale σ as hyperparameters (9): Jaccard, Bray–Curtis, unweighted UniFrac, and weighted UniFrac kernels.


(9) $$k(x,x^{\prime })=\sigma^{2}\;\text{exp}\;\left(-\frac{d{\left(x,x^{\prime }\right)}^{2}}{2l^{2}}\right),$$

In addition to these single-kernel functions, a multiple-kernel function was developed to integrate the information from multiple dissimilarity indices (10). Each single kernel within the multiple kernel has independent hyperparameters, specifically the length scale and the output scale.


(10) $$\begin{array}{c} k(x,x^{\prime })=k_{\text{JA}}(x,x^{\prime })+k_{\text{BC}}(x,x^{\prime })\\ \;+k_{\text{UU}}(x,x^{\prime })+k_{\text{WU}}(x,x^{\prime }) \end{array}.$$

### 2.3 Experiments

To evaluate the accuracy and reliability of the GP regression models using kernel functions based on microbial community dissimilarities, we used two publicly available 16S rRNA gene amplicon sequencing datasets of the human gut microbial communities. The first dataset was obtained from the American Gut Project (AGP), a large-scale citizen-scientist cohort (McDonald *et al.* 2018). The abundance table and phylogenetic tree were downloaded from the FTP site (ftp://ftp.microbio.me/AmericanGut/ag-2017-12-04/). We focused on the largest population (USA) and used a healthy adult subset as described by McDonald *et al.* (2018). This dataset was used for regression tasks predicting chronological age and body mass index (BMI). The second dataset was obtained from the IBD200 cohort (Mills *et al.* 2022), which is a study of patients with IBD. The abundance table and phylogenetic tree were downloaded from the Qiita platform (Gonzalez *et al.* 2018) with a study ID of 12675. We focused on Crohn’s disease patients and used this dataset for a regression task to predict disease severity, specifically the Crohn’s disease activity index (CDAI). For each dataset, OTUs/ASVs found in fewer than 1% of samples were excluded from further analysis. The details of the three regression tasks across the two datasets are presented in Table 1, and the distributions of the three target variables are shown in Supplementary Fig. S1.

**Table 1.** Summary of the datasets used in this study.

Target (dataset)	Number of samples	Number of OTUs/ASVs
Age (AGP; McDonald *et al.* 2018)	1639	4091
BMI (AGP; McDonald *et al.* 2018)	1639	4091
CDAI (IBD200; Mills *et al.* 2022)	104	1096

The relative abundance of each OTU/ASV was calculated by dividing each count by the total count per sample. The dissimilarity indices were then calculated. Jaccard and Bray–Curtis distances were calculated using the R package vegan (Dixon 2003), and unweighted and weighted UniFrac distances were calculated using the R package GUniFrac (Chen *et al.* 2012).

### 2.4 Implementations

The GP regression models were constructed using PyTorch (Paszke *et al.* 2019) and GPyTorch (Gardner *et al.* 2018). The hyperparameters of the GP models, including the length scale and output scale, were optimized through maximizing the marginal likelihood. The models were trained for 200 epochs with a learning rate of 0.1, with Adam (Kingma and Ba 2015) as the optimization function. All models were trained and tested with 10 repeated 50/50 split training and test sets.

The GP regression models were compared with other probabilistic prediction models, including NGBoost and deep ensembles. For NGBoost, we used the Python package NGBoost with default parameters. For deep ensembles, we employed both a phylogenetic CNN and a standard CNN. The phylogenetic CNN followed the same architecture as MDeep (Wang *et al.* 2021). MDeep first performs phylogeny-based clustering of OTUs and then captures phylogenetic correlations through a NN consisting of three one-dimensional convolutional layers and three fully connected layers. We modified the original MDeep to output the mean and variance of a normal distribution and trained it using maximum likelihood estimation. In contrast, the standard CNN had the same architecture but did not incorporate phylogeny-based clustering; instead, it used OTUs in shuffled order as input. Following Lakshminarayanan *et al.* (2017), we treated the ensemble as a uniformly weighted mixture model and combined the predicted distributions of the five models. The experimental settings for the CNN are included in the Supplementary Material.

### 2.5 Evaluation metrics

The generalization performance of the probabilistic prediction models was evaluated using the mean negative log likelihood (NLL) of the test data. NLL is widely used to assess model uncertainty on held-out data, with lower values indicating better performance. By measuring the uncertainty of predicted probability distributions based on observed data, NLL provides a reliable metric for probabilistic prediction. Given a predictive distribution $\hat{p}$, the NLL for N test samples was computed as follows:


(11) $$\text{NLL}=-\frac{1}{N}\sum_{i=1}^{N}\;\text{log}\;\hat{p}(y_{i}|x_{i},D),$$

where D represents the training data, and $x_{i}$ and $y_{i}$ denote the input features and target values in the test data, respectively. The accuracy of the mean values from the predictive distributions was assessed using the root-mean-square error (RMSE) as follows:


(12) $$\text{RMSE}=\sqrt{\frac{1}{N}\sum_{i=1}^{N}{\left(y_{i}-{\hat{y}}_{i}\right)}^{2}},$$

where ${\hat{y}}_{i}$ denotes the predicted mean value.

To evaluate the quality of predictive uncertainty estimation, we used both absolute and relative measures. As an absolute measure, we employed confidence-based calibration (Kuleshov *et al.* 2018, Gustafsson *et al.* 2020, Scalia *et al.* 2020), which means that an x% confidence interval should contain the true outcome approximately x% of the time. Specifically, we considered m confidence levels $0\leq p_{1}<p_{2}<\ldots <p_{m}\leq 1$ and computed the empirical coverage ${\hat{p}}_{j}$ at each threshold $p_{j}$. The quality of the calibration was quantified by the expected calibration error (ECE; see Supplementary Material). Additionally, the relationship between predictive uncertainty and accuracy was evaluated using a confidence curve and an oracle curve (Ilg *et al.* 2018, Scalia *et al.* 2020). This approach examines the variations in prediction error when data points with higher predictive uncertainty are sequentially removed from the test set. Ideally, the confidence curve is expected to exhibit a decreasing trend, indicating reduced prediction error when uncertain predictions are excluded. In contrast, the random removal of data points from the test set usually results in a flat, constant error curve. For comparison, the oracle curve was used to show the best decrease in prediction error, achieved by sequentially removing data points with the highest prediction error. The predictive uncertainty was quantified by the variances of the predictive distributions, and the prediction error was assessed using RMSE. The difference between the confidence curve and the oracle curve is quantified using the area under the confidence-oracle error (AUCO; see Supplementary Material).

## 3 Results

To quantify uncertainty in prediction, we constructed GP regression models using kernel functions based on multiple dissimilarity indices. First, we evaluated the effectiveness of these kernel functions and the predictive performance of the GP models in regression tasks for chronological age, BMI, and disease severity (CDAI). Next, we compared the quality of the predictive distributions with those of other probabilistic prediction methods. Finally, we assessed the relationship between estimated uncertainty and predictive accuracy.

### 3.1 Comparison of GP kernel functions

We developed four single-kernel functions based on microbial community dissimilarities: Jaccard, Bray–Curtis, unweighted UniFrac, and weighted UniFrac kernels. Additionally, a composite multiple-kernel function that combines these four single kernels was constructed. We compared the GP regression models using these different kernel functions.

The generalization performance of the GP regression models with different kernel functions was compared using NLL (Table 2). The results demonstrate that the GP model with the multiple-kernel function achieved the best NLL for all regression tasks. Compared with the commonly used linear and RBF kernels, the multiple-kernel GP model based on dissimilarity indices produced predictive distributions that more accurately fit the observed data. The multiple-kernel GP model consistently outperformed or performed comparably to the single-kernel GP models, indicating that the multiple-kernel approach effectively selected appropriate dissimilarities based on the observed data.

**Table 2.** Comparison of Gaussian process kernel functions using NLL and RMSE.^a^

	Age		BMI		CDAI	
Kernel function	NLL (↓)	RMSE (↓)	NLL (↓)	RMSE (↓)	NLL (↓)	RMSE (↓)
Linear	3.95 ± 0.00	12.48 ± 0.06	2.45 ± 0.00	2.80 ± 0.01	3.50 ± 0.03	**7.85** ± **0.20**
RBF	3.92 ± 0.01	12.24 ± 0.05	2.39 ± 0.00	2.64 ± 0.01	**3.43** ± **0.02**	7.92 ± 0.20
Jaccard	3.81 ± 0.01	**11.05** ± **0.06**	2.37 ± 0.00	2.60 ± 0.01	**3.44** ± **0.02**	**7.70** ± **0.15**
Bray–Curtis	3.85 ± 0.01	11.59 ± 0.07	**2.34** ± **0.00**	**2.54** ± **0.01**	**3.48** ± **0.03**	**7.74** ± **0.19**
Unweighted UniFrac	3.82 ± 0.01	**11.12** ± **0.06**	2.38 ± 0.00	2.62 ± 0.01	**3.47** ± **0.03**	7.88 ± 0.16
Weighted UniFrac	3.92 ± 0.00	12.23 ± 0.04	2.44 ± 0.00	2.77 ± 0.01	3.49 ± 0.03	**7.87** ± **0.18**
Multiple-kernel	**3.80** ± **0.01**	**11.02** ± **0.06**	**2.34** ± **0.00**	**2.53** ± **0.01**	**3.43** ± **0.04**	**7.50** ± **0.20**

a The means and standard errors of the means over 10 train-test splits are shown. The best method for each dataset is highlighted in bold, along with those whose standard errors overlap with it.

The predictive performance of different dissimilarity-based kernels varied across the datasets, in agreement with previous studies (Li *et al.* 2023; Xu *et al.* 2024). Among the four single-kernel functions, the Jaccard kernel achieved the best performance for chronological age prediction, whereas the Bray–Curtis kernel performed best for BMI prediction. This suggests that the dissimilarity indices contributing most to predictive performance differ depending on the target task. This variability highlights the importance of selecting task-specific kernels. Nevertheless, the multiple-kernel GP regression model integrates these indices by adjusting the kernel hyperparameters, thereby improving overall predictive performance.

The introduction of dissimilarity-based kernel functions improved the probabilistic prediction performance in all three regression tasks. The prediction performance was primarily assessed using NLL. Similar trends were observed for RMSE, although the models were not optimized for RMSE. We used the multiple-kernel GP regression model for comparison with other machine learning and deep learning methods and to evaluate the quality of uncertainty quantification.

### 3.2 Comparison of probabilistic prediction models

We then compared the probabilistic prediction performance of the multiple-kernel GP model with those of other probabilistic prediction models, including NGBoost and deep ensembles. NGBoost is a tree-based gradient boosting method that enables probabilistic regression. For deep ensembles, we used standard and phylogenetic CNNs as the NN architectures. The phylogenetic CNN has the same architecture as MDeep, which incorporates phylogenetic information.

The probabilistic prediction performance was evaluated using NLL on the test data. As a result, the multiple-kernel GP model outperformed existing methods across the three regression tasks, achieving the lowest NLL (Table 3). This superiority was particularly evident in the regression of CDAI. Deep ensembles achieved lower NLL than NGBoost, although there was not much difference in performance between the two architectures of the deep ensembles (i.e. the phylogenetic CNN and the standard CNN). This suggests that incorporating phylogenetic information into the CNN architecture did not enhance the probabilistic prediction performance in this context.

**Table 3.** Comparison of predicted probability distributions using NLL.^a^

Method	Age	BMI	CDAI
NGBoost	4.20 ± 0.02	2.73 ± 0.01	628.45 ± 213.06
Deep ensembles (standard CNN)	3.96 ± 0.01	2.48 ± 0.00	3.58 ± 0.06
Deep ensembles (phylogenetic CNN)	3.97 ± 0.01	2.48 ± 0.01	3.58 ± 0.05
Multiple-kernel GP model	**3.80** ± **0.01**	**2.34** ± **0.00**	**3.43** ± **0.04**

a The means and standard errors of the means over 10 train-test splits are shown. The best method for each dataset is highlighted in bold, along with those whose standard errors overlap with it.

To evaluate the mean values of the predictive distributions, we compared the multiple-kernel GP model with several point prediction methods, including ridge, lasso, elastic net, random forests, and multi-kernel machine regression (MKMR; Li *et al.* 2023). MKMR utilizes multiple kernels and optimizes their weights but does not adjust the internal hyperparameters of the kernels, such as the length scale. For MKMR, we employed the same combination of kernels as used in our multiple-kernel GP model for comparison. As shown in Supplementary Table S1, we observed that the GP model offers superior or comparable point prediction performance compared with these methods, including random forests, although our method was not specifically optimized for point prediction.

### 3.3 Evaluation of uncertainty estimates

**Figure 1.**
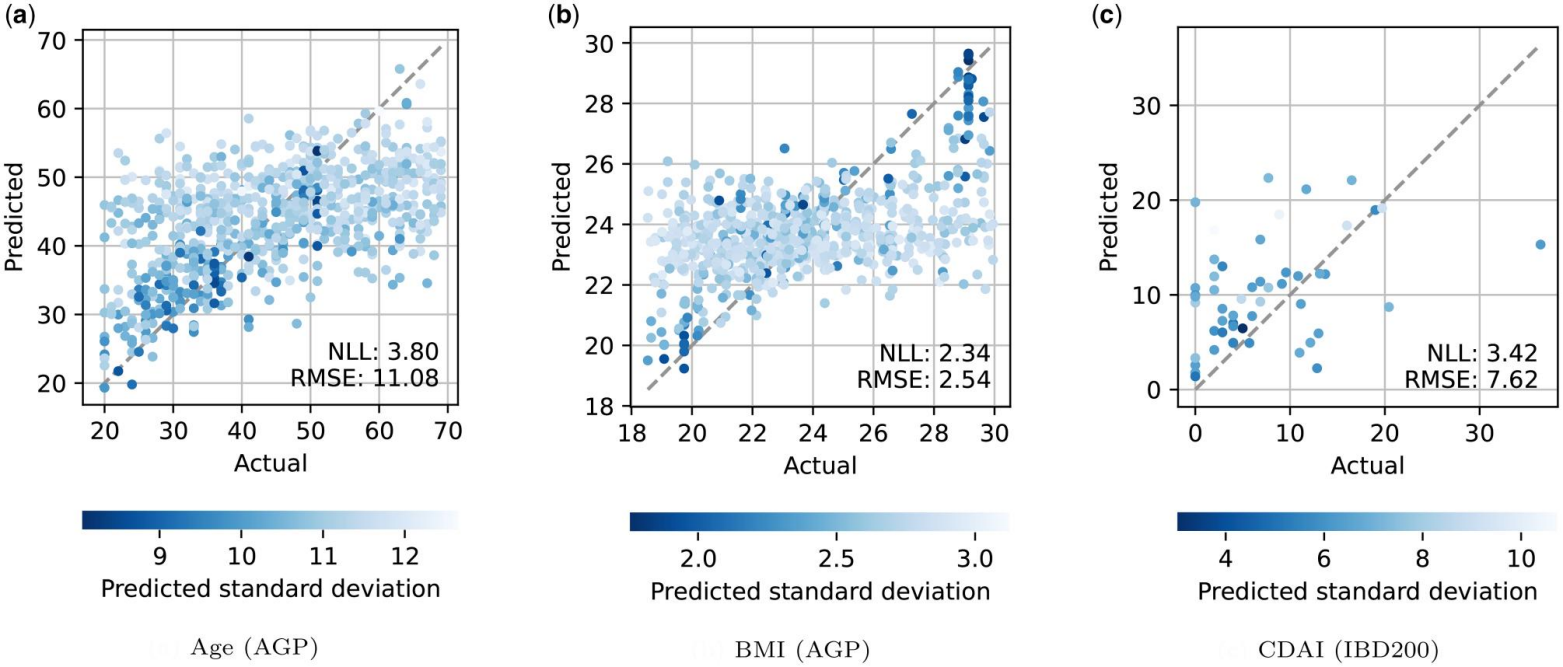
Scatter plots of predicted versus actual values for the regression tasks of (a) age, (b) BMI, and (c) CDAI. The predicted values represent the means of the predictive probability distributions, with colors indicating their standard deviations. Each plot corresponds to the train-test split where NLL was closest to the mean across 10 train-test splits.

The scatter plots of actual versus predicted values using the multiple-kernel GP models are shown in Fig. 1. We observed that data points with higher predictive accuracy tended to have smaller predicted standard deviations, indicating more reliable predictions. In the age prediction task, predictions for samples from younger individuals tended to be more confident. In the BMI prediction task, samples from individuals with either significantly lower or higher BMI values were predicted with greater confidence. These heterogeneous distributions of predictive uncertainty may contribute to a more comprehensive biological understanding and the detection of temporal shifts in the human microbiome.

**Figure 2.**
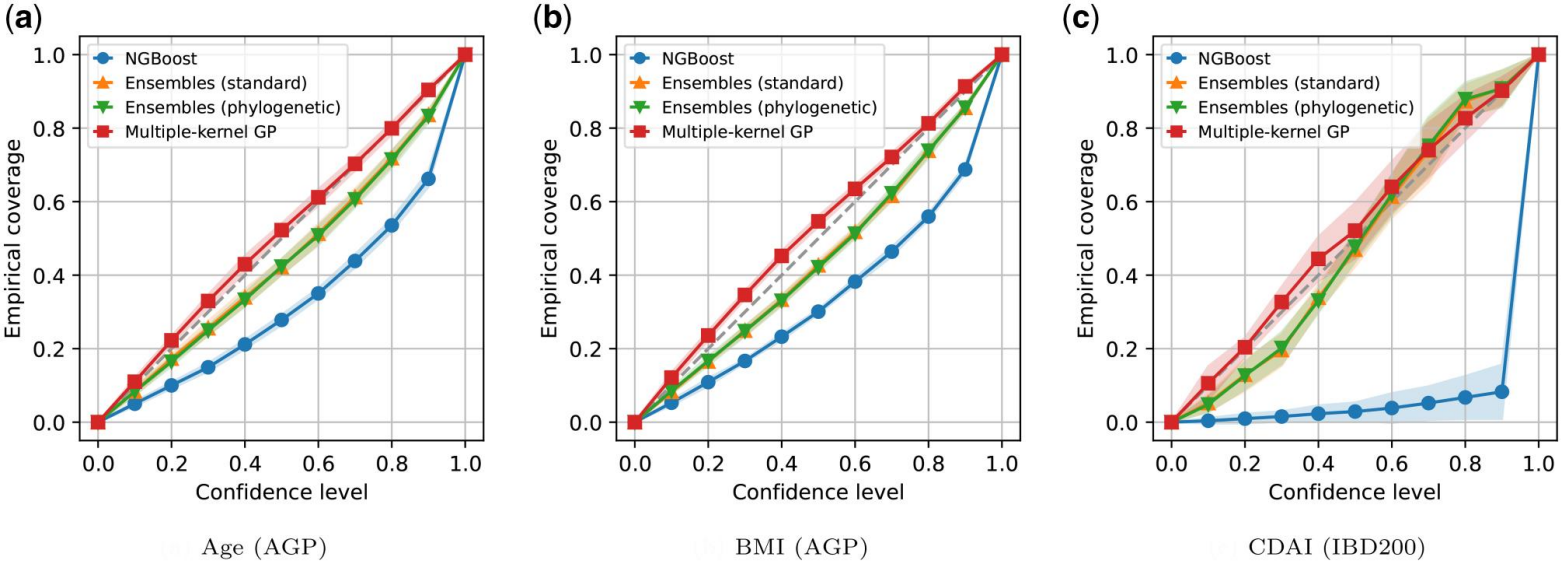
Calibration plots for probabilistic regression models across three tasks: (a) age, (b) BMI, and (c) CDAI. The empirical coverage at each confidence level is shown. Solid lines represent the means, and shaded regions represent standard deviations across 10 train-test splits. Gray dashed lines represent perfect calibration.

As an absolute measure for evaluating predictive uncertainty, we used the calibration error based on confidence intervals. As shown in the calibration plots (Fig. 2), the confidence level and the empirical coverage closely matched in multiple-kernel GP regression models, whereas other methods tended to be below the diagonal. Indeed, the multiple-kernel GP model demonstrated superior calibration performance in terms of ECE (Table S2).

**Figure 3.**
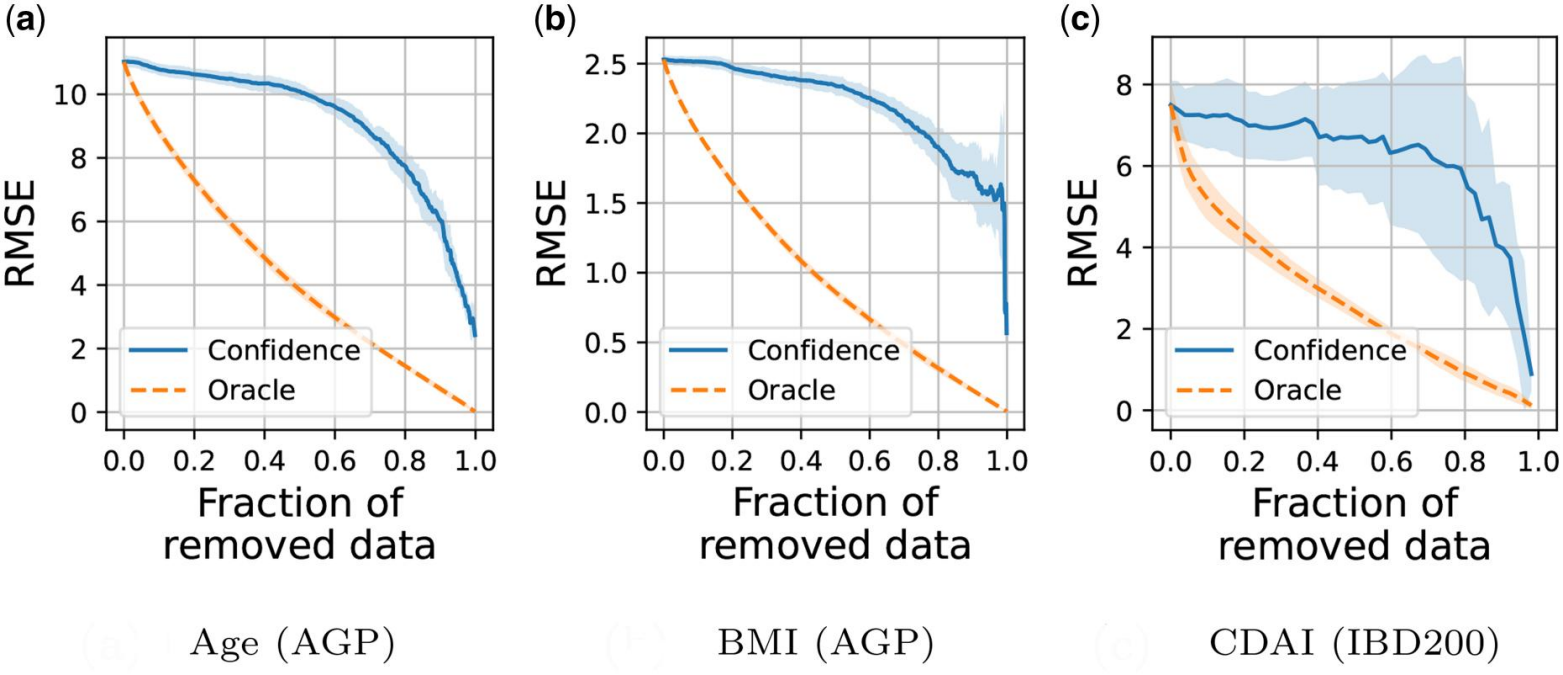
Confidence and oracle curves for multiple-kernel Gaussian process regression models across three tasks: (a) age, (b) BMI, and (c) CDAI. The confidence curves show that prediction errors decreased when data points with higher predictive variance were sequentially removed from the test set. The oracle curves represent the optimal removal of data points with the largest errors. Solid lines represent the means, and shaded regions represent standard deviations across 10 train-test splits.

The relationship between predictive uncertainty and prediction error was assessed using confidence and oracle curves, as shown in Fig. 3. The confidence curves demonstrate that prediction error decreases as highly uncertain data points are removed across all three datasets, suggesting a close association between predictive uncertainty and prediction error. The oracle curves, which represent the optimal error reduction by removing the data points with the largest errors, show a nearly linear decrease. Compared with the oracle curves, the slope of the confidence curves in the initial (left) half is more gradual across all three datasets. In contrast, in the latter (right) half, the confidence curves demonstrate a more pronounced decrease. When examining the top 10% most confident predictions, we found that the mean prediction error decreased to ∼50%–60% of the original prediction error. The confidence curves of other methods are shown in Supplementary Figs S2–S4. Compared with other methods, the multiple-kernel GP model matched or outperformed them in terms of AUCO (Supplementary Table S3).

## 4 Discussion

This study presents a method to improve predictive uncertainty estimation in microbiome-based models by incorporating microbial community dissimilarities into the GP kernel function. The proposed model enhances uncertainty quantification while maintaining or improving predictive accuracy compared with existing machine learning methods. It leverages the strengths of GP models in handling high-dimensional and sparse microbial abundance data, as well as their flexibility in integrating microbial phylogenetic information. These advantages make the method robust across various prediction tasks and suitable for applications requiring accurate uncertainty quantification.

For clinical applications, it is crucial to establish criteria to determine when to use or refrain from using model predictions (Kompa *et al.* 2021). For individuals with highly uncertain predictions, additional experiments may be needed to obtain more accurate information. Because we found that model confidence is positively related to prediction performance, predictive uncertainty can provide a reliable threshold. This allows for flexibility in setting confidence thresholds based on the clinical requirements and acceptable error margins. Our improved uncertainty quantification supports better-informed healthcare decisions and represents a significant step toward more reliable microbiome-based prediction, potentially advancing personalized medicine.

We used a combination of multiple dissimilarity indices as kernel functions for the GP models. Combining multiple dissimilarity indices is commonly used in microbiome analyses. For example, MiRKAT (Zhao *et al.* 2015) and PERMANOVA-S (Tang *et al.* 2016) used multiple dissimilarity indices to statistically test the association between microbial diversity and covariates of interest, whereas MKMR (Li *et al.* 2023) and MK-BMC (Xu *et al.* 2024) were developed as kernel prediction models for microbiome data. These studies have demonstrated that combining multiple dissimilarity indices effectively captures the complexity and diversity of microbiome data. Giliberti *et al.* (2022) showed that the presence of specific OTUs is more important than their relative abundance for some host phenotype prediction tasks, emphasizing the advantage of using qualitative dissimilarity indices. Additionally, incorporating other dissimilarity indices not used in this study, such as the distance in the PhILR transformed space (Silverman *et al.* 2017), is possible. Taken together, the combination of multiple dissimilarity indices is beneficial in microbiome research, and our approach enhances the flexibility of this integration by optimizing two hyperparameters: the length scale and the output scale.

Chronological age, BMI, and disease severity (CDAI) were used as target variables in the regression models. Previous studies have developed predictive models for estimating chronological and biological age (Huang *et al.* 2020, Galkin *et al.* 2020, Seo *et al.* 2023), predicting obesity (Xu *et al.* 2024), and assessing disease severity (Mills *et al.* 2022). Thus, utilizing these target variables provides a reliable basis for comparing the performance of different machine learning models. These models are expected to enhance our understanding of how variations in the human microbiome are influenced by factors such as age, BMI, and disease progression. We observed that predictive uncertainty was heterogeneously distributed across the range of each target variable, particularly chronological age. Our findings suggest that older microbial communities exhibit greater variability, potentially reflecting individual-specific patterns of dysbiosis associated with aging and age-related disorders. Although microbial changes during aging remain a topic of debate—one study reported a decrease in diversity (Aleman and Valenzano 2019), whereas another indicated an increase in specific age-associated microbial taxa (Badal *et al.* 2020)—our results highlight greater variability in older microbial communities than in younger ones.

One limitation of microbiome-based predictive models using GPs is their scalability. Adapting GP models to large-scale datasets is challenging because of the time complexity of $O(N^{3})$ and the storage requirement of $O(N^{2})$, where N is the number of samples. A recent review by Dudek *et al.* (2024) reported that many microbiome datasets consist of hundreds to thousands of samples. Although GP models work well for these small to medium-sized datasets, they encounter computational challenges in large-scale microbiome studies. To address this limitation when dealing with larger microbiome datasets, computationally scalable GP variants, such as sparse GPs (Titsias 2009), are promising. These methods approximate the exact GP using a subset of inducing points, substantially reducing the computational complexity while maintaining much of the predictive power of the exact GP.

## 5 Conclusion

In this study, we developed probabilistic prediction models using GPs to predict host characteristics such as age, BMI, and disease severity from human microbial abundance data. Our approach integrates microbial community dissimilarities into a kernel function, enabling more effective modeling of phylogenetic, high-dimensional, and sparse microbial abundance data. The model demonstrated superior performance in quantifying predictive uncertainty compared with existing methods. The robustness of our model across various regression tasks suggests its broad applicability in clinical settings. Future research should explore its potential for daily health monitoring and early disease detection. This study represents an important step toward more reliable microbiome-based prediction models for clinical applications.

## Supplementary Material

vbaf045_Supplementary_Data

## Data Availability

The code is available at https://github.com/asahiadachi/gp4microbiome.
