# Supplementary material for: Quantifying uncertainty in microbiome-based prediction using Gaussian processes with microbial community dissimilarities
Source: Bioinform Adv. 2025 Mar 11;5(1):vbaf045. doi: 10.1093/bioadv/vbaf045 (PMC11919817; doi:10.1093/bioadv/vbaf045)
Supplement: vbaf045_Supplementary_Data [file vbaf045_supplementary_data.pdf]

Supplementary Material for “Quantifying uncertainty in microbiome-based prediction using Gaussian processes with microbial community dissimilarities”

Asahi Adachi, Fan Zhang, Shigehiko Kanaya and Naoaki Ono

1 Methods

1.1 Experimental settings for CNN models

We adopted the CNN architecture described in Wang et al. (2021). We implemented probabilistic prediction by modifying the network to output the mean and variance of a Gaussian distribution. Following Lakshminarayanan et al. (2017), we used the negative log-likelihood as the loss function. We trained the networks with a batch size of 16 for 2000 epochs, using the Adam optimizer (Kingma and Ba, 2015) with a learning rate of 0.0001. For deep ensembles, we trained the networks five times using different random seeds. Experiments were conducted using PyTorch.

1.2 Expected calibration error (ECE)

We evaluated uncertainty using the confidence-based calibration (Kuleshov et al., 2018; Gustafsson et al., 2020; Scalia et al., 2020). Given m confidence levels $0 \leq p_1 < p_2 < \cdots < p_m \leq 1$, the empirical coverage $\hat{p}_j$ at each threshold p_j is computed as follows.

$$\hat{p}_j = \frac{1}{T} \sum_{t=1}^{T} \mathbb{I} \left\{ F_t^{-1} \left(\frac{1 - p_j}{2} \right) \leq y_t \leq F_t^{-1} \left(\frac{1 + p_j}{2} \right) \right\}, \tag{1}$$

where F_t is the predicted cumulative distribution function for sample t, and T is the number of samples. A calibration plot shows $\hat{p}_j$ versus p_j. If the model is perfectly calibrated, this plot follows a straight line. To quantify the degree of calibration, we use the ECE given by

$$\mathrm{ECE} = \frac{1}{m} \sum_{j=1}^{m} |p_j - \hat{p}_j| . \tag{2}$$

In this study, we use the thresholds $p_j = 0, 0.1, \ldots, 0.9, 1.0$. A smaller ECE indicates better calibration, with 0 corresponding to perfect calibration.

1.3 Area under the confidence-oracle error (AUCO)

We quantified the difference between the confidence curve and the oracle curve using the AUCO (Ilg et al., 2018; Scalia et al., 2020). Specifically, for each $i = 1, 2, \ldots, N-1$, we removed i samples from the test set according to either the confidence-based ranking or the ideal (oracle-based) ranking, and then measured the RMSE on the remaining subset. The AUCO is defined as

$$\mathrm{AUCO} = \sum_{i=1}^{N-1} \left(\mathrm{RMSE}_i^{(\mathrm{confidence})} - \mathrm{RMSE}_i^{(\mathrm{oracle})} \right), \tag{3}$$

where $\mathrm{RMSE}_i^{(\mathrm{confidence})}$ and $\mathrm{RMSE}_i^{(\mathrm{oracle})}$ are the respective errors on the subset after removing i samples. A smaller AUCO value indicates that the confidence-based ranking more closely approximates the ideal (oracle-based) ranking.

1.4 Comparison of point prediction performance

We compared the point prediction performance of the multiple-kernel GP model with that of several point prediction methods, including ridge, lasso, elastic net, random forests, and MKMR (Li et al., 2023). For ridge, lasso, elastic net, and random forests, we used the Python library scikit-learn (Pedregosa et al., 2011). We tuned the regularization parameters for ridge, lasso, and elastic net using five-fold cross-validation over the search range provided by scikit-learn. We set "max_iter" to 5000 for lasso and elastic net, and "l1_ratio" to {0.1, 0.5, 0.7, 0.9, 0.95, 0.99, 1} for elastic net. All other parameters were set to their default values. For MKMR, we used the original publicly available MATLAB implementation[1].

2 Supplementary Figures

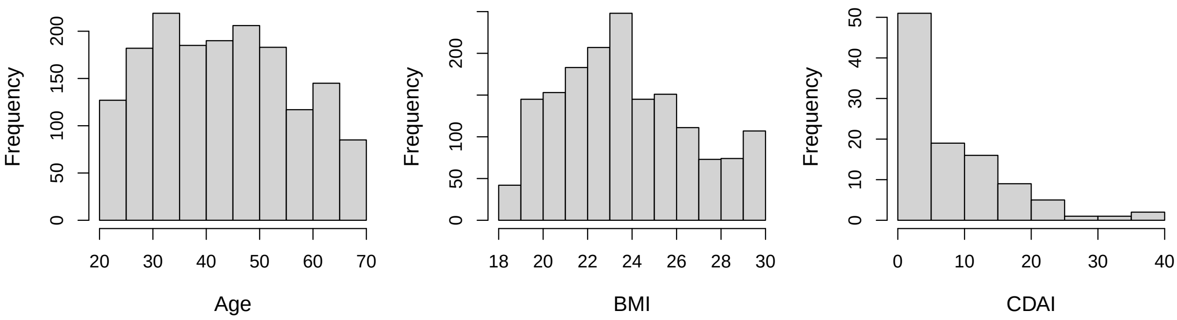

(a) Age (AGP) (b) BMI (AGP) (c) CDAI (IBD200)

Figure S1. Histograms of the target variables used in this study: (a) chronological age, (b) body mass index (BMI), and (c) Crohn's disease activity index (CDAI).

[1]https://github.com/BingLi17/MKMR

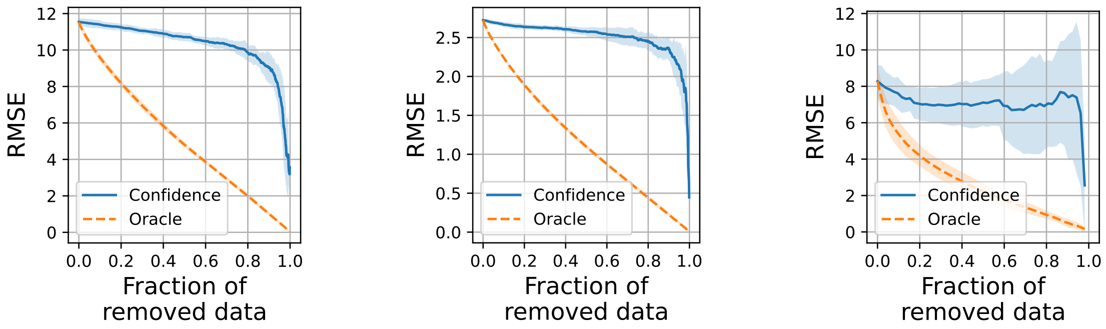

(a) Age (AGP) (b) BMI (AGP) (c) CDAI (IBD200)

Figure S2. Confidence and oracle curves for NGBoost.

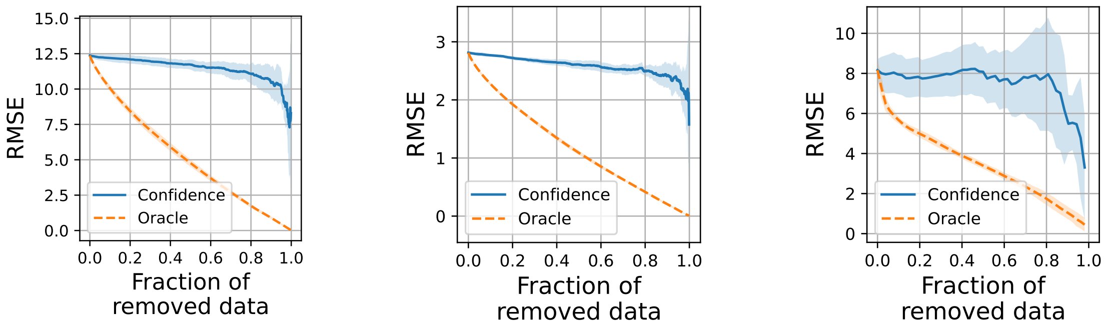

(a) Age (AGP) (b) BMI (AGP) (c) CDAI (IBD200)

Figure S3. Confidence and oracle curves for deep ensembles (standard CNN).

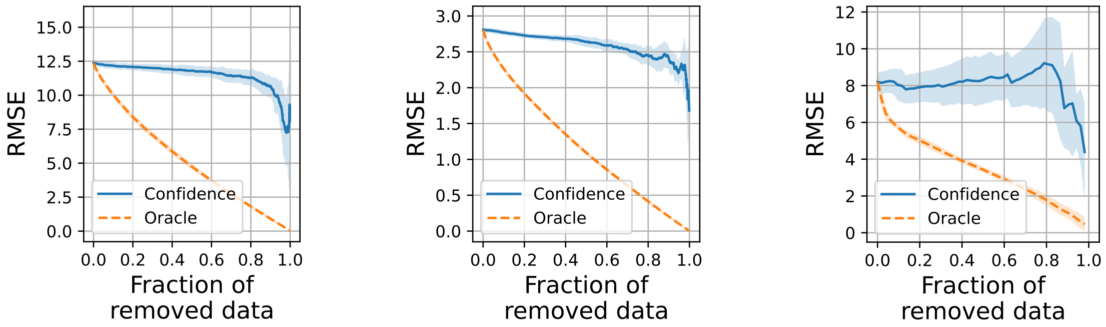

(a) Age (AGP) (b) BMI (AGP) (c) CDAI (IBD200)

Figure S4. Confidence and oracle curves for deep ensembles (phylogenetic CNN).

3 Supplementary Tables

Table S1. Comparison of point prediction performance using RMSE. The means and standard errors of the means over 10 train-test splits are shown. The best method for each dataset is highlighted in bold, along with those whose standard errors overlap with it.

Method	Age	BMI	CDAI
Ridge	12.44 ± 0.06	2.79 ± 0.01	**7.63 ± 0.17**
Lasso	12.53 ± 0.07	2.79 ± 0.01	**7.74 ± 0.15**
Elastic net	12.50 ± 0.07	2.80 ± 0.01	**7.73 ± 0.21**
Random forests	11.50 ± 0.06	2.68 ± 0.01	**7.88 ± 0.22**
MKMR	11.23 ± 0.06	2.70 ± 0.01	**7.57 ± 0.18**
Multiple-kernel GP model	**11.02 ± 0.06**	**2.53 ± 0.01**	**7.50 ± 0.20**

Table S2. ECE results for probabilistic regression models across three regression tasks. The means and standard errors of the means over 10 train-test splits are shown. The best method for each dataset is highlighted in bold, along with those whose standard errors overlap with it.

Method	Age	BMI	CDAI
NGBoost	0.157 ± 0.003	0.140 ± 0.002	0.380 ± 0.008
Deep ensembles (standard CNN)	0.050 ± 0.004	0.047 ± 0.002	0.052 ± 0.005
Deep ensembles (phylogenetic CNN)	0.053 ± 0.004	0.047 ± 0.003	0.054 ± 0.003
Multiple-kernel GP model	**0.017 ± 0.002**	**0.026 ± 0.002**	**0.038 ± 0.007**

Table S3. AUCO results for probabilistic regression models across three regression tasks. The means and standard errors of the means over 10 train-test splits are shown. The best method for each dataset is highlighted in bold, along with those whose standard errors overlap with it.

Method	Age	BMI	CDAI
NGBoost	4302.5 ± 64.9	1099.4 ± 9.1	227.0 ± 24.0
Deep ensembles (standard CNN)	5199.8 ± 102.6	1161.9 ± 14.2	**209.9 ± 18.6**
Deep ensembles (phylogenetic CNN)	5211.4 ± 95.3	1166.3 ± 15.9	235.7 ± 18.0
Multiple-kernel GP model	**3921.5 ± 42.2**	**1003.9 ± 22.8**	**180.2 ± 19.1**

References

Gustafsson, F. K. et al. (2020). Evaluating Scalable Bayesian Deep Learning Methods for Robust Computer Vision. In *Proceedings of the IEEE/CVF Conference on Computer Vision and Pattern Recognition Workshops*, pages 318–319.

Ilg, E. et al. (2018). Uncertainty Estimates and Multi-Hypotheses Networks for Optical Flow. In *Proceedings of the European Conference on Computer Vision (ECCV)*, pages 652–667.

Kingma, D. P. and Ba, J. (2015). Adam: A Method for Stochastic Optimization. In *International Conference for Learning Representations.*

Kuleshov, V. et al. (2018). Accurate Uncertainties for Deep Learning Using Calibrated Regression. In *Proceedings of the 35th International Conference on Machine Learning*, pages 2796–2804. PMLR.

Lakshminarayanan, B. et al. (2017). Simple and Scalable Predictive Uncertainty Estimation using Deep Ensembles. In *Advances in Neural Information Processing Systems*, volume 30. Curran Associates, Inc.

Li, B. et al. (2023). MKMR: A multi-kernel machine regression model to predict health outcomes using human microbiome data. *Briefings in Bioinformatics*, 24(3):bbad158.

Pedregosa, F. et al. (2011). Scikit-learn: Machine Learning in Python. *Journal of Machine Learning Research*, 12(85):2825–2830.

Scalia, G. et al. (2020). Evaluating Scalable Uncertainty Estimation Methods for Deep Learning-Based Molecular Property Prediction. *Journal of Chemical Information and Modeling*, 60(6):2697–2717.

Wang, Y. et al. (2021). A novel deep learning method for predictive modeling of microbiome data. *Briefings in Bioinformatics*, 22(3):bbaa073.
